# Discrete constriction locations describe a comprehensive range of vocal tract shapes in the Maeda model

**DOI:** 10.1121/10.0009058

**Published:** 2021-12-28

**Authors:** Jessica L. Gaines, Kwang S. Kim, Benjamin Parrell, Vikram Ramanarayanan, Srikantan S. Nagarajan, John F. Houde

**Affiliations:** 1 Graduate Program in Bioengineering, University of California Berkeley-University of California San Francisco, California 94143, USA; 2 Department of Otolaryngology-Head and Neck Surgery, University of California, San Francisco, California 94143, USA; 3 Department of Communication Sciences and Disorders, University of Wisconsin–Madison, Madison, Wisconsin 53715, USA; 4 Modality.AI, San Francisco, California 94105, USA; 5 Department of Radiology and Biomedical Imaging, University of California, San Francisco, California 94143, USA jessica.gaines@berkeley.edu, kwangseob.kim@ucsf.edu, bparrell@wisc.edu, vikram.ramanarayanan@ucsf.edu, srikantan.nagarajan@ucsf.edu, jfhoude@ucsf.edu

## Abstract

The Maeda model was used to generate a large set of vocoid-producing vocal tract configurations. The resulting dataset (a) produced a comprehensive range of formant frequencies and (b) displayed discrete tongue body constriction locations (palatal, velar/uvular, and lower pharyngeal). The discrete parameterization of constriction location across the vowel space suggests this is likely a fundamental characteristic of the human vocal tract, and not limited to any specific set of vowel contrasts. These findings suggest that in addition to established articulatory-acoustic constraints, fundamental biomechanical constraints of the vocal tract may also explain such discreteness.

## 1. Introduction

Speech production requires numerous muscles to coordinate articulators within highly demanding spatiotemporal constraints. Previous work in speech motor control has postulated that such a complex and challenging process may be efficiently controlled by high-level task goals defined in terms of the location and size (or “degree”) of constrictions in the vocal tract (e.g., Browman and Goldstein, 1992; Saltzman and Munhall, 1989). This idea that speech production may control vocal tract constrictions, rather than the positions of the individual articulators, has been supported by both experimental data and results from computational modeling. For example, studies found that when a mechanical perturbation is applied to an articulator (e.g., jaw), compensatory responses are found in both perturbed and non-perturbed articulators that are task-relevant to the given speech sound (e.g., Abbs and Gracco, 1984; Abbs *et al.*, 1984; Kelso *et al.*, 1984; Shaiman, 1989). On the other hand, perturbations to articulators that are not task-relevant do not cause compensatory responses (Shaiman and Gracco, 2002).

From a computational perspective, the TADA (Task Dynamics Application, Nam *et al.*, 2004) model, which incorporates the CASY (Configurable Articulator Synthesizer, Iskarous *et al.*, 2003) plant, has demonstrated that the activation of dynamically defined constriction tasks can successfully simulate speech movements and acoustics. High quality acoustic results of speech synthesis based on the control of vocal tract constrictions have been shown using a more realistic vocal tract model by Birkholz *et al.* (2006). Recently, the FACTS (Feedback Aware Control of Tasks in Speech, Parrell *et al.*, 2019) model—whose architecture combines control laws governed by Task Dynamics with principles of State Feedback Control for estimating the state of the body—showed that constriction-based task control can simulate online compensation responses to unexpected auditory feedback perturbations. Despite these successes, how vocal tract constrictions are specified in the speech motor system remains largely unexamined. Task Dynamics, as originally formulated, posited a single task of “tongue body constriction location” that determined the location of vocalic constrictions in the vocal tract, with the implicit assumption that these constrictions could be produced anywhere along the (post-alveolar) vocal tract.

However, there is some evidence that human speakers make use of a more limited set of constriction locations. From the perspective of speech perception, when Iskarous *et al.* (2010) asked participants to listen to [ɑɪ] sequences that were generated by CASY and give naturalness ratings to the sounds, the participants reported high naturalness ratings for the sounds generated with discrete constriction locations compared to sequences with more continuous changes in constriction location. From the perspective of speech production, quantal theory, as proposed by Stevens (1989), suggests that regions along the vocal tract vary in the amount of change in acoustics that is associated with small changes in constriction location. The author concludes that areas that have low sensitivity to small modulations in tongue placement are better suited for constrictions during speech, to allow for imprecision in vocal control. Similar acoustic sensitivity responses were also reported by Mrayati *et al.* (1988). Speech data also provides evidence of discrete constriction locations during production of individual vowels across different languages. For example, in a review of X-ray data from 40 subjects speaking 13 languages, Wood (1979) found four constriction locations across subjects, listed as the hard palate, soft palate, upper pharynx, and lower pharynx. Mokhtari *et al.* (2007) and Story and Titze (1998) developed empirical models of vocal tract shape based on data from Japanese and English vowels, respectively. Although common constriction locations were not explicitly noted, some constriction locations shared between vowels can be seen in the vocal tract area function data. Finally, Boë *et al.* (1992) estimated vocal tract configurations for 10 French vowels using simulations with the Maeda vocal tract model (Maeda, 1990). Although the intent was to characterize each vowel independently, the aggregated results suggest these constrictions cluster in a limited set of locations.

Despite the lingual diversity of the data discussed above, each of the aforementioned studies is limited to a subset of the F1–F2 space. Most examined distinct vowel categories in specific languages. Some also examined the transition space between a few vowels (Mokhtari *et al.*, 2007; Story and Titze, 1998), but none ventured to span the whole space of possible F1–F2 combinations. Thus, it remains unclear whether this is a unique characteristic of vowel categories *per se*, or rather a more general property of vocal tract shaping during speech production. Examining constriction parameters in a dataset spanning the full range of possible vocoid sounds can provide additional insights. Rather than considering only the acoustic-to-articulatory mappings, such analyses may reveal the biomechanical constraints on acoustically relevant constrictions in the vocal tract.

The present study, therefore, applied the Maeda model to explore a comprehensive range of vocoid-producing configurations. Although there are certainly other means of examining the vocal tract constrictions such as real-time MRI data, the Maeda model has been used previously as a sufficiently accurate approximation of vocal tract shape and acoustic data (e.g., Ouni and Laprie, 2005; Potard *et al.*, 2008). Here, we focused primarily on constrictions posterior to the alveolar ridge given that vocoids are mainly determined by constrictions in the tongue body. Critically, our dataset of approximately 1 × 10^6^ vocoid-producing vocal tract shapes was not limited to individual vowels in any particular language, but densely covered the F1–F2 space. Examining the location and degree of vocal tract constrictions in this dataset allows for the examination of patterns in vocal tract constrictions outside of and beyond individual vowels. This study therefore builds on the ideas of quantal theory of speech by examining the output space of the empirical Maeda model.

## 2. Methods

Developed based on cineradiographic data, the Maeda model can generate two-dimensional midsagittal vocal tract contours (i.e., vocal tract shapes) and their corresponding formant frequencies based on seven input parameters (Maeda, 1990). Each of the input parameters is maximally independent from one another based on a guided principal component analysis (PCA) and corresponds closely with one of the following: jaw position (“jaw”), tongue dorsal position (“tongue”), the arched or flattened shape of the tongue (“shape”), the raised or lowered position of the tongue apex (“apex”), the height of the upper lip above the lower lip (“lip height”), the distance between the minimum lip height and the upper incisors (“lip protrusion”), and the height of the larynx (“larynx”). Given that the parameters were determined by PCA, the value of each parameter is defined in terms of standard deviations above or below the mean value.

The source code for the Maeda model was obtained from the Vocal Tract model GitHub repository (Ghosh, 2013). The source code, originally written in C, was wrapped with Python 3.6 (Van Rossum & Drake, 2009) using an open-source software tool, Simplified Wrapper and Interface Generator (SWIG, Beazley, 1996). The resulting Python-wrapped code was called from a custom-built Python script that generated arbitrary vocal tract configurations by varying six of the seven Maeda input parameters (the “larynx” parameter was held constant at zero, since this parameter has a relatively little impact on the formant frequencies).

In order to densely sample the space of vocal tract configurations that correspond to vocoid production, a random walk approach was employed to sample the six free Maeda input parameters. At the starting point of each random walk, a value between -3 and 3 was drawn from a uniform random distribution and assigned to each Maeda input parameter. As noted earlier, each input parameter is in units of standard deviations from the mean, and the range from −3 to 3 covers the vast majority of speech sounds (Maeda, 1990). The Maeda model was then used to map this input vector to the corresponding vocal tract shape and formant values. For each subsequent step, a random step value between −0.25 and 0.25, drawn from a uniform distribution, was added to each input parameter value from the previous point (i.e., random walk). This step range (from −0.25 to 0.25) was determined to provide adequate density in the data and distance to explore the space. Because the intent was to sample only those configurations that correspond to vocoid production, two criteria were verified at each step: (1) the formant synthesis resulted in a full set of formant values (F1–F5), and (2) F1 had a value between 250 and 900 Hz.^1^ Initial testing suggested that vocal tract configurations with consonant-like (close or extremely narrow constrictions) failed to generate five formants (F1–F5) or resulted in extremely high F1 values. If the given formant output of the model did not fit the two criteria, the random walk was stopped, and a new random walk process was initiated. Each random walk was limited to a maximum of 50 steps to avoid oversampling the space adjacent to each starting point and to reduce the dataset's dependence on the initialization of each random walk.

**Fig. 1.**
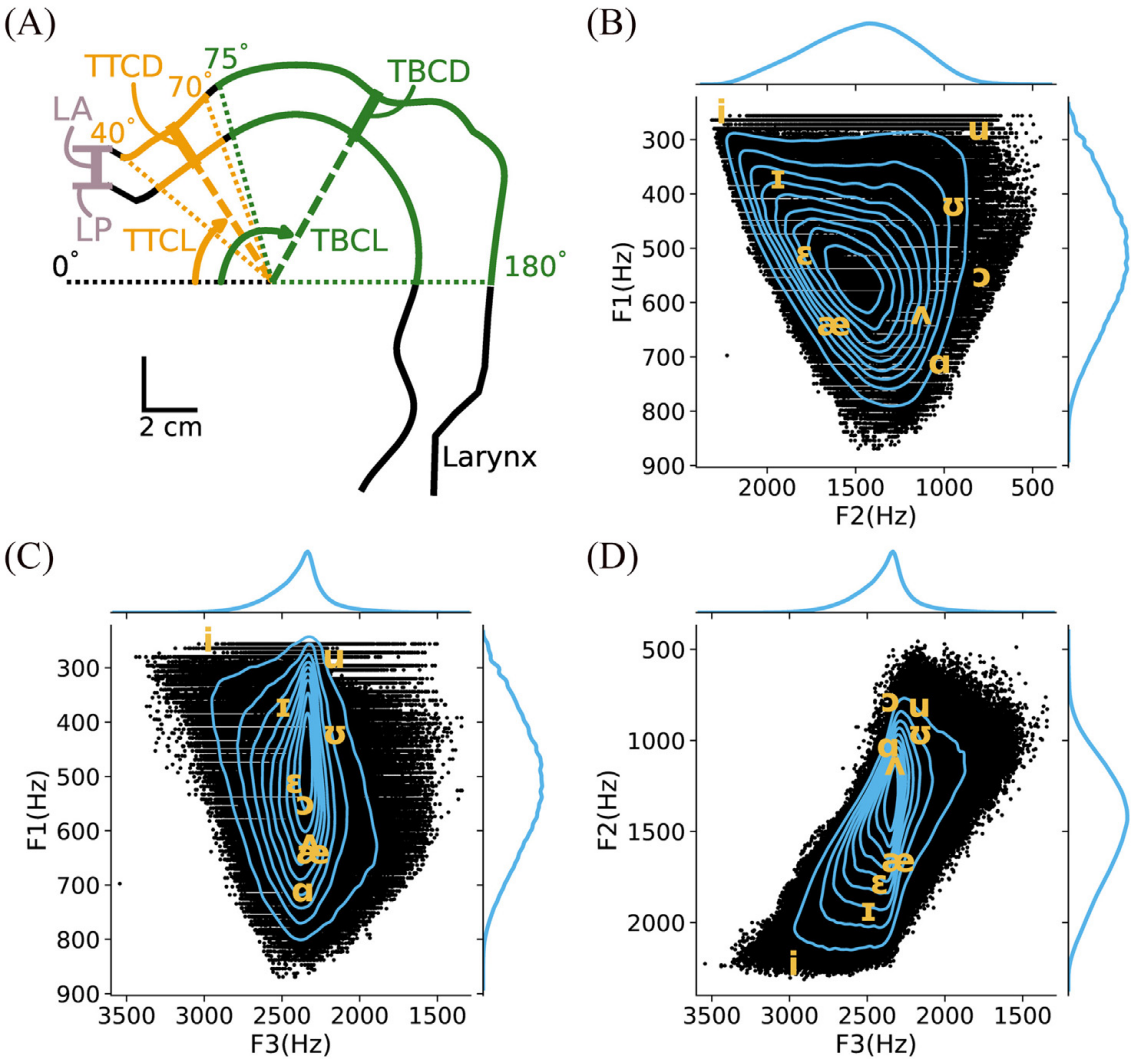
(A) Task parameters: lip aperture (LA), lip protrusion (LP), tongue tip constriction location (TTCL), tongue tip constriction degree (TTCD), tongue body constriction location (TBCL), and tongue body constriction degree (TTCD). (B)–(D) The formants produced by the valid vocal tract shapes cover the F1–F2 (B), F1–F3 (C), and F2–F3 (D) spaces. For reference, average formant frequencies of American English vowels for male speakers are shown in the IPA symbols (Peterson and Barney, 1952).

The random walk algorithm was executed until it saved 1 200 000 configurations. For each configuration, six constriction-based task parameters were calculated: lip aperture (LA), lip protrusion (LP), tongue tip constriction location (TTCL), tongue tip constriction degree (TTCD), tongue body constriction location (TBCL), and tongue body constriction degree (TTCD) [Fig. 1(A)]. These constriction-based task parameters have been extensively used and tested in various models (e.g., Nam *et al.*, 2004; Parrell *et al.*, 2019). First, LA and LP were determined using Maeda's vocal tract shape output (i.e., two lists of 29 [x,y] points in a sagittal Cartesian plane defining the inner and outer boundaries of the vocal tract). LA was calculated as the difference between the y-coordinate values of the most anterior points of the inner vocal tract shape and the outer vocal tract shape. LP was calculated as the difference between the x-coordinate values of the two most anterior points of the inner vocal tract shape [see Fig. 1(A)].

To determine the remaining task parameters, an origin point was set at the center of the Maeda's vocal tract shape space ([x,y] position of [0,0], roughly corresponding to the center of the tongue) so that it could be used as the origin of a polar grid. Next, the resolution of each vocal tract shape (which is originally 29 [x,y] points) was increased by fitting cubic spline and re-sampling the vocal tract contour, resulting in 29 602 [x,y] points for the outer vocal tract trace (i.e., pharyngeal wall, palate and upper lip) and 50 000 for the inner vocal tract trace (i.e., tongue surface and lower lip). Next, to create the polar grid, 1401 lines were drawn outward from the origin shown in [Fig. 1(A)], every 0.1° from 40°–180° clockwise from the left horizontal. This range spans from the upper teeth to the middle of the oropharynx. The inner vocal tract point and outer vocal tract point closest to each polar grid line were selected, resulting in 1401 angle-matched pairs of [x,y] points in each trace. Thus, the distance between the inner vocal tract trace and the outer vocal tract trace could be measured at each 0.1°. The tongue tip constriction parameters were defined in the region from 40°–70° clockwise from the left horizontal. Tongue tip constriction degree (TTCD) was defined as the minimum distance between the inner and outer vocal tract in the region (in millimeters), and tongue tip constriction location (TTCL) was the angle at which this minimum distance was found. The tongue body constriction location (TBCL) and degree (TBCD) were defined in the same manner in the region from 75°–180° clockwise from the left horizontal.

Of the 1 200 000 vocal tract configurations, 192 946 (about 16% of the dataset), had tongue body or tongue tip constriction degrees that were less than 5 mm. As very narrow constrictions of this type are likely to result in frication rather than the laminar airflow of typical vocoid production (Boë *et al.*, 1992), these were removed from the dataset, leaving 1 007 104 vocal tract configurations. The results presented below based on this reduced dataset were broadly similar for the full dataset.

## 3. Results

The random walk algorithm generated a dataset of approximately 10^6^ vocal tract shapes, along with their corresponding formant frequencies and task parameters. The formants produced by the vocoid-producing vocal tract configurations in this set covered a wide range of the F1–F2 space [see Fig. 1(B)]. The distribution was approximately bell-shaped with highest density near the median values for both F1 and F2. The F1–F3 [Fig. 1(C)] and F2–F3 spaces [Fig. 1(D)] were also well covered by this dataset. The average formant frequencies for male speakers reported by Peterson and Barney (1952) were well within the range of this dataset, providing further evidence that the dataset contained a near-complete set of vocoid-producing configurations.

**Fig. 2.**
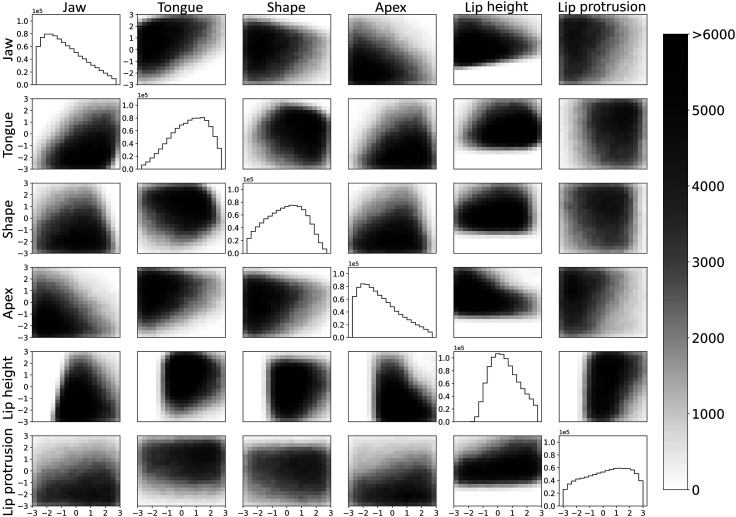
Prevalence of value combinations of Maeda input parameters in vocoid-producing vocal tract shapes, as indicated by two-dimensional histograms across pairs of Maeda parameters. Darker regions indicate a higher prevalence of observations. The one-dimensional histograms for each Maeda input parameter are shown on the diagonal.

The prevalence of Maeda input parameters represented in this dataset can be seen in Fig. 2. Each off-diagonal plot shows a 2D histogram of vocoid-producing vocal tract configurations across a pair of Maeda parameters. One-dimensional histograms for each Maeda input parameter are shown in the plots on the diagonal, and often resemble skewed bell curves. Since the random walk algorithm drew values from a uniform distribution, an over-representation of a parameter value or combination of values in the dataset indicates a parameter range favorable to producing vowel-like sounds. Parameter ranges under-represented in the dataset indicate that these inputs often led to non-vocoid configurations. For example, negative values of tongue dorsal position (“tongue”), which correspond with positions closer to the front of the mouth, often produced non-vocoid configurations when combined with negative values of tongue shape (“shape”), which correspond with a flatter tongue. When the tongue is flat, it is likely to collide with the alveolar ridge at more frontal positions, leading to an invalid vocal tract shape for vowel production. As another example, negative values of “lip height”, which correspond with smaller height of the upper lip above the lower lip, are very rare in vocoid-producing vocal tract configurations. Thus, there are much lighter regions on the negative side of all plots related to lip height, and essentially no vocoid-producing configurations in the negative region of the one-dimensional histogram for lip height.

**Fig. 3.**
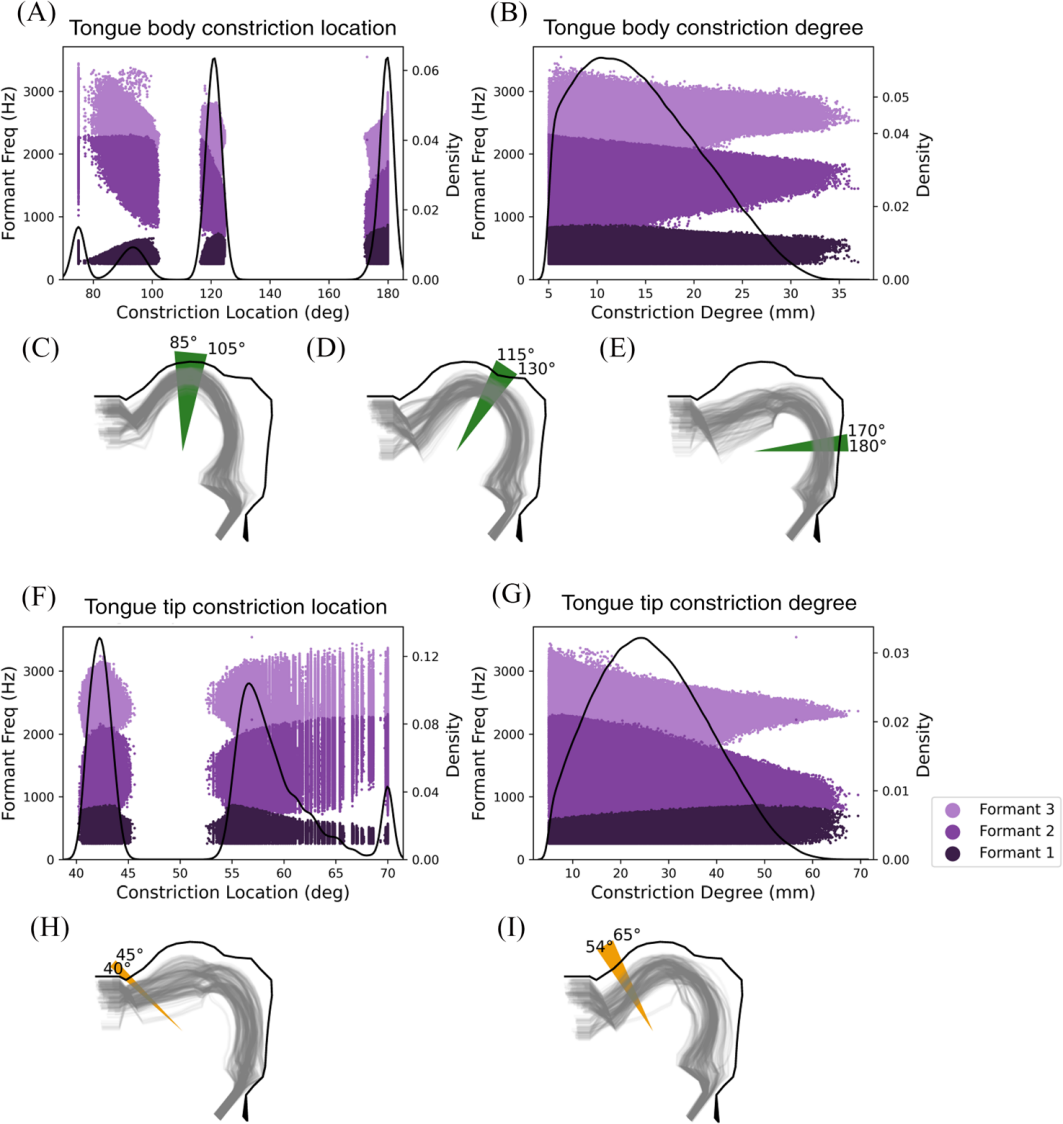
The density of observations across TBCL (A) and TBCD (B), along with the associated range of F1 (dark purple), F2 (medium purple), and F3 (light purple). For each of the three discrete TBCL ranges, a subset of 500 randomly chosen vocal tract shapes are shown in green (C)–(E). Also shown are the density of observations of TTCL (F) and TTCD (G) and their associated ranges of F1, F2, and F3. A random subset of 500 vocal tract shapes are also shown from each of the two discrete TTCL ranges (H)–(I).

Although the distributions of Maeda input parameters and their corresponding formant frequencies were unimodal and approximately bell-shaped (albeit with varying degrees of skewedness), the distributions of the corresponding task parameters calculated from the same configurations did not follow this pattern. Rather, the values of constriction locations represented in the dataset were limited to a few discrete ranges, as seen in Fig. 3. TBCL exhibited a trimodal distribution, showing that constriction in the tongue body was restricted to locations around 95° (palatal), 120° (velar/uvular), or 180° (pharyngeal) from the horizontal axis [see Fig. 3(A)]. A random subset of vocal tract shapes characterized by each of these constriction locations is shown in Figs. 3(C)–3(E). TTCL showed a bimodal distribution with greater density around 40°–45° (dental) and 55°–65° (alveolar). Figures 3(H) and 3(I), respectively, illustrate a random subset of the shapes containing constrictions in each of these locations.

Notably, Figs. 3(A) and 3(F) show a high density of vocal tract shapes with constrictions at the very boundary of the defined tongue tip and tongue body ranges (i.e., anterior palatal region around 70° or 75°). However, we do not consider the anterior palatal region to be a constriction location in the tongue body or the tongue tip, because these peaks in density are an artifact of our defined boundary between tongue body and tongue tip. Nearly all of the observations in the 70° and 75° peaks indicate vocal tract shapes that narrow as the boundary is approached, and continue to narrow beyond the boundary. For these vocal tract shapes, the constriction location must always be at the boundary region, no matter where the boundary is located. Thus, when the boundary between tongue body and tongue tip was temporarily redefined to another location, a cluster of observations formed at the new boundary, but no cluster was present in the 70°–75° region. The density peaks at the anterior end of Fig. 3(A) (tongue body constriction location) and the posterior end of Fig. 3(F) (tongue tip constriction location) are therefore artifacts and not true constriction locations. Additional comments regarding data clusters at the locations 70° and 75° will thus be omitted from subsequent discussions of these results.

**Fig. 4.**
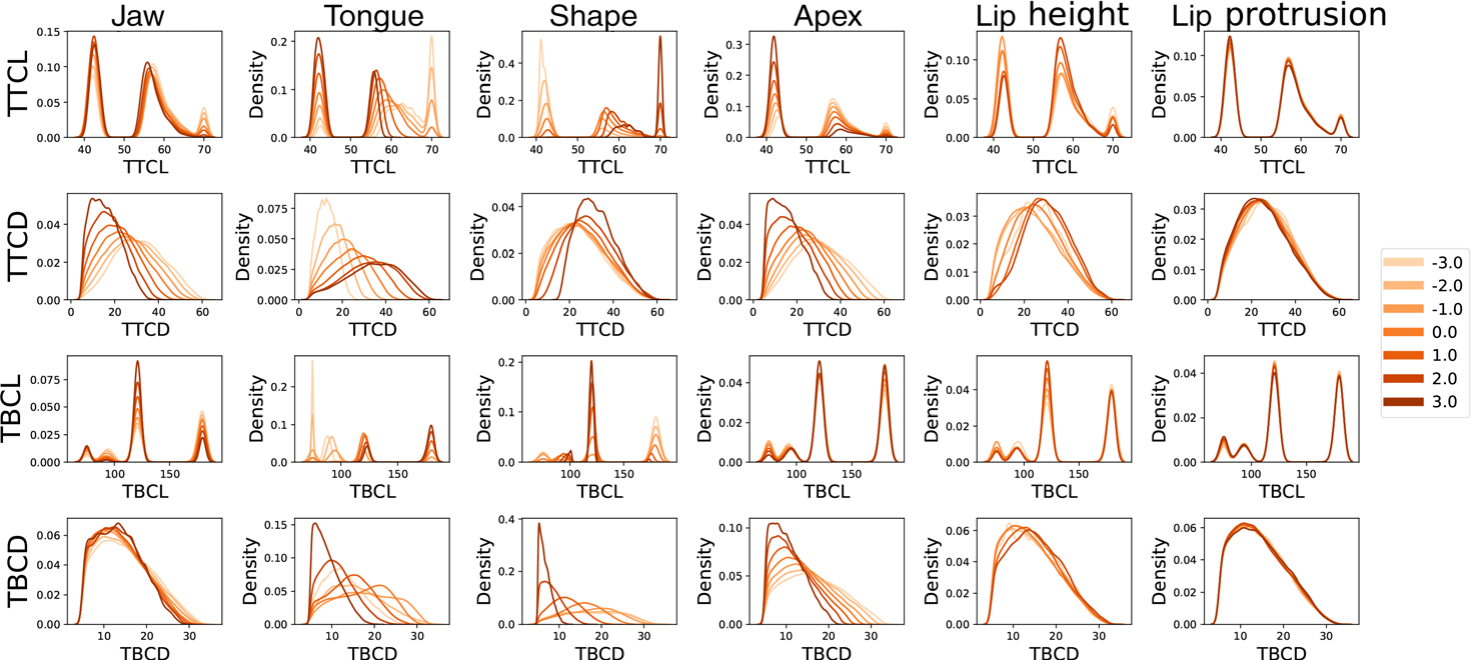
Covariation of task parameters with Maeda parameters. Each subplot shows the density of vocoid-producing configurations across a given task parameter for seven different ranges of values for each Maeda parameter. Each colored curve within each subplot corresponds to the subset of vocal tract shapes with a different Maeda parameter value, ±0.5. Lighter orange curves indicate more negative Maeda values, while darker orange curves indicate more positive values.

The Maeda input parameters that describe vocal tract shape have a complex, many-to-one relationship with each task parameter. Figure 4 describes these relationships by showing the variation in the distribution of task parameter density for different values of each Maeda parameter. The bi- and tri-modal distributions of TTCL and TBCL seen in Fig. 3 were also found in the covariation of task parameters with individual Maeda parameters; however, the relative density of observations at each constriction location often changed based on the value of an associated Maeda input parameter. For example, the Maeda parameter for the flattened or arched shape of the tongue (“shape”) corresponded strongly with constriction location (see Fig. 4, Shape). A more arched tongue shape (more positive value, shown in dark orange) corresponded strongly with the velar/uvular constriction location in the tongue body, around 120° from the horizontal axis. A more flattened tongue shape (more negative value, shown in light orange) corresponded strongly with a pharyngeal constriction (around 180°) in the tongue body and a dental constriction (40°–45°) in the tongue tip. Finally, the alveolar constriction location in the tongue tip (55°–65°) and the palatal location in the tongue body (around 95°) tended to be characterized most often by a more neutral tongue shape with moderate arch (bright orange).

It can also be seen that the Maeda parameter for tongue dorsal position (“tongue”) varied strongly with TBCL. A more anterior position of tongue (more negative value, shown in light orange) corresponded to smaller TBCL, composing a high proportion of vocal tract shapes with a palatal constriction. Interestingly, for the tongue tip, a more anterior “tongue” value was associated with a more posterior TTCL near the alveolar ridge. This phenomenon is consistent with tongue movement, because a constriction near the pharynx or velum allows greater freedom in tongue tip movement, while a constriction near the palate is too close to the tongue tip region to allow independent movement, and necessarily creates a constriction in the most adjacent area of the tongue tip.

Finally, a more anterior tongue dorsal position (more negative value of “tongue”, shown in light orange) corresponded with smaller constriction degree in the tongue tip, while a more posterior tongue dorsal position (more positive value, shown in dark orange) corresponded with greater range in TTCD. This indicates that a more posterior tongue dorsal position provides greater independence to the degree of tongue tip constrictions as well their location. In contrast, Maeda parameters that were not associated with given task parameters showed similar density distributions across all values of the Maeda parameter (e.g., see lip height or lip protrusion with TBCL in Fig. 4).

## 4. Discussion

In this study, a large dataset of vocoid-producing vocal tract configurations was generated using a random walk algorithm through Maeda model input parameter space. The generated dataset covered a comprehensive set of vocoid productions, as evidenced by the fact that the range of formant values produced by the dataset covered the range typically reported in the literature (e.g., Peterson and Barney, 1952). Although unimodal distributions characterized both the input parameter space and the resulting formants, these same data in the task space were restricted to three discrete constriction locations in the tongue body, seen as a multimodal density distribution for TBCL.

The current study notes the finding of Boë *et al.* (1992)—discrete tongue constriction locations in a Maeda-generated dataset for 10 French vowels—and extends it to a dataset of approximately 10^6^ vocoid-producing vocal tract shapes that span the F1–F2 space, not restricted to any specific language or vowel set. It should be noted that while Boë *et al.* (1992) found a total of two discrete constriction locations, the current study found three constriction locations in the tongue body region (and an additional two in the tongue tip region). This discrepancy may be due to methodological differences in measuring constriction locations. In the Boë *et al.* (1992) study, constrictions were measured from the whole vocal tract and quantified in distance along the vocal tract, whereas the current study measured constrictions separately in the tongue tip and tongue body, and used angular measurements of constriction location. Nevertheless, the discrete constriction locations found in both Boë *et al.* (1992) and this extended dataset suggest that this discreteness may be a more general property of human speech rather than characteristics of a specific language or certain vowels.

Our findings of discrete constriction locations are consistent with the quantal theory (Stevens, 1989), which suggests that due to acoustic stability, there is a probability distribution along the length of the vocal tract of how likely each location is to form a constriction. The present results build on this theory to suggest that in the Maeda model, an empirical model guided by articulatory movements, this probability distribution manifests as a set of discrete constriction locations. This implies that over and above acoustic stability constraints, biomechanical constraints may also play a role in determining where constrictions occur in the vocal tract. This idea is in line with the results of Perrier *et al.* (2000), who found evidence of physiological constraints based on their experiments with principal components analysis of the movements of a two-dimensional, biomechanical tongue model. Thus, the presence of discrete constriction locations may be explained by the biomechanics of the vocal tract and not only by articulatory–acoustic stability relationships.

It is important to note the limitations of this study. First, the dataset explored in this study was not collected from human speakers but was instead generated using the Maeda model of the vocal tract. The data may therefore contain biases associated with the model. For example, the Maeda model is based on still-frame cineradiographic data and therefore, does not reflect the kinematics of a moving vocal tract. In addition, the cineradiographic data were extracted from 10 French sentences produced by two French speakers. It is therefore possible that the characteristics of task variable space explored in this study may not apply to all of human speech, but may instead apply specifically to the French language, the particular sentences used for data collection, or the subjects whose speech was analyzed. However, the data's coverage of the F1–F2 space shows that a comprehensive range of vocoid sounds are represented, which suggests these results are not confined to a particular language. For example, despite the French origins of the Maeda model data, average male formant values for American English vowels (Peterson and Barney, 1952) are well within the formant frequency ranges generated in this study.

Another limitation is that our data simulate only one individual speaker given that speaker-specific characteristics of the vocal tract (e.g., vocal tract size) were kept constant in this study. In some versions of Maeda model, the mouth and pharynx scaling factors can be altered to fit the model to articulatory data collected from different speakers, as has been done with magnetic resonance (MR) image data by Mathieu and Laprie (1997) and Potard and Laprie (2010); x-ray image data by Panchapagesan and Alwan (2011); and electromagnetic articulography (EMA) data by Toutios and Narayanan (2013). Therefore, future studies are warranted to confirm our finding across multiple speakers using readily available MR, x-ray, and EMA data.

Additionally, even though the dataset covered the F1–F2 space, it was certainly not a complete set of vocal tract shapes found in speech. First, in the random walk algorithm used to generate vocal tract shapes, only six parameters were varied. While varying six principal components of the Maeda dataset was expected to cover most of the variation, some extreme shapes may have been missing from the dataset due to excluding some components. Specifically, the dataset was restricted by fixing the “larynx” Maeda parameter at zero. Although the physical articulator of the larynx does not affect formant values in speech, the Maeda parameter labeled “larynx” is not a pure representation of larynx movement, but a PCA component of the data that corresponded well with larynx length. Thus, adjusting the parameter results in changes in the shape, length, and cross section area of the laryngeal portion of the vocal tract, which affect formant frequencies. However, it is important to note that such changes result in only small alterations in formant frequencies. For example, from the neutral position (all Maeda input parameters are set to 0), decreasing the larynx parameter from 0.0 to –2.0 results in a 9 Hz decrease in F1, which is only about 2% change from the original F1 (453 Hz). Increasing the larynx parameter from 0.0 to 2.0 results in a 10 Hz increase in F1 (about 2.2% change). To confirm that the “larynx” parameter being fixed at 0 did not affect our results, the analysis was repeated with data generation in which the “larynx” parameter was allowed to vary. Because the changes in formant frequencies were small in amount, the findings were essentially unaffected by its removal. Lastly, the dataset was restricted to only vocoid-producing vocal tract configurations, so any configurations expected to produce consonant-like sounds were not represented. This may especially affect this study's findings regarding constrictions in the tongue tip, since the tongue tip is primarily used to produce consonant sounds. Since this was a study of vocoid-producing vocal tract shapes, the focus was on tongue body constrictions. The tongue tip constrictions shown here should be considered a distribution of tongue tip constrictions that correspond with the given tongue body constrictions during vocoid productions.

## 5. Conclusion

This study investigated how varying the parameters of the Maeda vocal tract model affected the location and size of vocal tract constrictions at the tongue body and tongue tip. Using random walks through the Maeda model input parameter space, a near-complete set of vocoid-producing vocal tract shapes was generated that covered all of the F1–F2 vowel space. Importantly, the locations of tongue body and tongue tip constrictions corresponding with these shapes were found to be restricted to discrete ranges of values at the teeth, alveolar ridge, hard palate, velum, and lower pharynx. This implies that the large range of vocal tract shapes generated by randomly varying six Maeda features can be reduced to three discrete constriction locations produced by the tongue body and two produced by the tongue tip. This discreteness is not limited to particular linguistic vowel categories (Boë *et al.*, 1992; Wood, 1979), but is rather a more general property of vocoid-producing vocal tract shapes, and thus may arise from fundamental biomechanical constraints of the vocal tract in addition to established articulatory–acoustic relationships.

## Footnotes

^1^
The F1 range was determined to allow a wider range than the average male F1 range across American English vowels (Peterson and Barney, 1952). The F1 range for male speakers was used as reference because even though the initial version of the Maeda model was developed based on the vocal tract images of two female French speakers, it was later transformed to reflect the vocal tract length and formant values of a male speaker.
